# Early Microvascular and Oscillatory Potentials Changes in Human Diabetic Retina: Amacrine Cells and the Intraretinal Neurovascular Crosstalk

**DOI:** 10.3390/jcm10184035

**Published:** 2021-09-07

**Authors:** Edoardo Midena, Tommaso Torresin, Evelyn Longhin, Giulia Midena, Elisabetta Pilotto, Luisa Frizziero

**Affiliations:** 1 Department of Neuroscience—Ophthalmology, University of Padova, 35128 Padova, Italy; tommaso.torresin@gmail.com (T.T.); evelyn.longhin@unipd.it (E.L.); elisabetta.pilotto@unipd.it (E.P.); lfrizziero@gmail.com (L.F.); 2 IRCCS—Fondazione Bietti, 00198 Rome, Italy; giulia.midena@gmail.com

**Keywords:** diabetic retinopathy, optical coherence tomography angiography, oscillatory potentials, amacrine cells, morpho-functional correlations, intraretinal crosstalk, electroretinography, vascular plexuses

## Abstract

To analyze the early microvascular retinal changes and oscillatory potentials alterations secondary to diabetic retinal damage, 44 eyes of 22 diabetic patients without and with mild diabetic retinopathy (DR) and 18 eyes of 9 healthy controls were examined. All subjects underwent spectral domain optical coherence tomography (SD-OCT), OCT angiography (OCTA), and electroretinography of oscillatory potentials (OPs). At OCTA, vessel area density (VAD), vessel length fraction (VLF), and fractal dimension (FD) were significantly reduced in the superficial vascular plexus (SVP), VLF and FD in the intermediate capillary plexus (ICP), and FD in the deep capillary plexus (DCP) in the diabetic group compared to the control group. The amplitude (A) of OP2, OP3, OP4 and the sum of OPs were significantly reduced in the diabetic group versus the controls, and the last two parameters were reduced also in patients without DR versus the controls. Moreover, in the diabetic group, a significant direct correlation was found between the A of OP1, OP2, OP3 and sOP and the VLF and FD in the SVP, while a statistically significant inverse correlation was found between the A of OP3 and OP4 and the VDI in the ICP and DCP. The reduced oscillatory potentials suggest a precocious involvement of amacrine cells in diabetic eyes, independently of DR presence, and their correlation with vascular parameters underlines the relevance of the crosstalk between these cells and vascular components in the pathophysiology of this chronic disease.

## 1. Introduction

Diabetes mellitus currently represents one of the emerging threats to public health all over the world, and diabetic retinopathy (DR) is a cause of visual impairment and blindness in the world [1]. Retinal microvascular damage has been considered the main pathophysiological driver of retinal damage induced by diabetes, including endothelial cell damage, pericyte loss, and secondary breakdown of the blood–retinal barriers [2]. However, some studies have shown that a systemically impaired glucose metabolism causes early dysfunction in the neural retina [3]. Even if an impairment of neurovascular coupling in the retina of patients with diabetes has been reported even before clinically visible DR, the crosstalk among vessels, neurons, and glial cells in the retina is still partly unknown, as well as its contribution to retinal damage secondary to diabetes [3,4,5,6].

With the development of optical coherence tomography angiography (OCTA), microvascular changes have been non-invasively visualized and quantified even in diabetic eyes without DR [7,8,9]. However, we still need to investigate the correlation between these new morphological findings and retinal function parameters [10,11]. Electroretinography (ERG) has shown to detect early neuronal abnormalities, before clinically detectable DR [10,12,13]. We previously reported that different microvascular and functional alterations, detected by means of OCTA and multifocal electroretinogram, may be found in diabetic patients at different early retinal clinical stages [14]. Oscillatory potentials (OPs), subcomponents of flash ERG, have been reported as one of the most sensitive functional parameters in diabetic eyes, clinically useful also to predict the rate of progression of retinopathy [12,15,16]. Moreover, they have been suggested to reflect disturbances in retinal circulation [16].

The aim of this study was to evaluate and correlate both structural and vascular retinal changes—obtained by means of OCT and OCTA, respectively—with oscillatory potentials in diabetic eyes without and with early clinical signs of DR, compared to healthy controls.

## 2. Materials and Methods

This was a cross-sectional observational study with a prospective enrolment. Diabetic patients without or with mild DR (only microaneurysms) according to the Clinical Diabetic Retinopathy Disease Severity Scale referred from November 2018 to February 2019, were consecutively recruited [17]. Age-matched healthy subjects were also enrolled, as a control group. The main ocular exclusion criteria were: history of glaucoma or ocular hypertension, any intraocular disease other than DR, proliferative DR, presence of macular edema, refractive error >3 diopters, any intraocular surgery in the last six months, any concomitant topical treatment (except for artificial drops), history of retinal laser treatment, ocular media opacities precluding the quality of retinal imaging. The main systemic exclusion criteria were: presence of any neurodegenerative disease or nervous system disorders not related to diabetes, any uncontrolled systemic diseases, recent (3 months) Hba1c ≥ 9%. All patients underwent a complete ophthalmic examination, including refraction and best-corrected distance visual acuity (BCVA) measurement, anterior segment examination, indirect ophthalmoscopy, 90-D lens biomicroscopy. They also underwent spectral-domain OCT, OCTA, and electroretinography of oscillatory potentials (OPs). An informed consent was obtained from each patient, and the research was carried out in accordance with the Declaration of Helsinki.

### 2.1. Imaging

OCT and OCTA were performed using the Spectralis HRA + OCT2 platform (Heidelberg Engineering, Heidelberg, Germany), as previously described [14].

Briefly, an automatic segmentation software (segmentation technology; Heidelberg Engineering, version 6.3.1.0) was used to identify and measure the thickness of retinal single layers in the 9 sectors of the ETDRS map. After automated segmentation, manual refinement was eventually performed in case of errors or artefacts. Mean thickness of each layer was considered for the study (mean value of the 4 quadrants of both inner and outer rings; the central 1 mm-diameter ring was considered as total mean thickness), as previously described [14].

As regards OCTA, the automatic segmentation algorithm Heyex Software 6.9a automatically detected the superficial vascular plexus (SVP) from the retinal nerve fiber layer (RNFL) to 17 μm above the inner plexiform layer [IPL− (IPL− corresponds to 17 μm above IPL, as automatically provided by the device)], the intermediate capillary plexus [ICP, from IPL− to IPL+ (IPL+ corresponds to 22 μm below IPL)], and the deep capillary plexus (DCP, from IPL+ to OPL). Measurement of the foveal avascular zone (FAZ) was performed manually, using the incorporated software tool, in the SVP, ICP, and DCP, automatically calculated in mm^2^.

Quantitative analysis of the vascular plexuses in the OCTA en-face images was performed using the open-source available ImageJ software (National Institutes of Health, Bethesda, MD, USA). To quantify each bi-dimensional en-face image, four parameters were analyzed: vessel area density (VAD), vessel length fraction (VLF), vessel diameter index (VDI), and fractal dimension (FD) [14]. A masked operator (TT) performed all OCTA measurements.

### 2.2. Oscillatory Potentials

ERG OPs were recorded according to the International Society for Clinical Electrophysiology of Vision (ISCEV) standards using a Roland RETI-port/scan 21 instrument (Roland Consult, Brandemburg, Germany) [18]. The exam was performed after pupil dilation (1% tropicamide eye drops) in both eyes. Disposable fiber DTL electrodes (DTL plus, Diagnosis LLC, Lowell, MA, USA) were used as active electrodes, while skin electrodes were positioned, after skin cleansing with an abrasive gel, near each orbital rim as a reference. The ground skin electrode was positioned on the mastoid. Before performing the exams, all patients were dark-adapted for 20 min. The light stimulus for the OPs recording was a standard flash of 3.0 cd⋅s⋅m^−2^ with a frequency of 0.1 Hz. The flash stimulus was generated in a Ganzfeld stimulator (ColorDome; Diagnosys LLC, Lowell, MA, USA), and the amplifier band pass was set at 100–300 Hz. The amplitude (A) of OP1, OP2, OP3, and OP4 was measured from trough to peak. The peak times (T) from stimulus onset to the peak were also noted for each OP. Additionally, the summed amplitude of the four OPs (sOP) was calculated for each eye. A single masked operator (EL) performed all OP measurements.

### 2.3. Statistical Analysis

The study variables were summarized according to the usual methods provided by descriptive statistics: mean values and standard deviation for the quantitative variables, absolute and relative (percentage) frequency for the qualitative ones.

OCT, OCTA, and OP parameters were analyzed by means of two-way ANOVA with interaction, for repeated measures: groups represented the “between” factor; plexuses (superficial, intermediate, and deep) represented the “within” factor; group by plexus represented the interaction factor. Comparison between patients and controls were made for each plexus.

Correlation analysis between vascular and oscillatory potentials parameters was performed among patients. For this aim, linear regression models were applied: a regression coefficient was used as a measure of correlation, and a statistical test on that coefficient as inference about the significance of such correlation.

All models were adjusted for replication of measurements on both eyes of the same subject.

Statistical analyses were performed by SAS^®^ v.9.4 statistical software (SAS Institute, Cary, NC, USA); statistical tests were interpreted as significant if *p* < 0.05.

## 3. Results

Forty-four eyes of 22 Caucasian diabetic patients (diabetic group), 11 without and 11 with mild DR, and 18 eyes of 9 healthy volunteers (control group) were included in the study. Best corrected visual acuity was 85 ETDRS score in all studied eyes. The main characteristics of the study population are summarized in Table 1. For all parameters, no statistically significant difference between the groups was found.

### 3.1. OCT and OCTA Parameters

The mean thickness of all single retinal layers analyzed both in the central 6 mm of the macular area and in the 1 mm-diameter central circle did not significantly differ between the groups [14].

**Figure 1:**
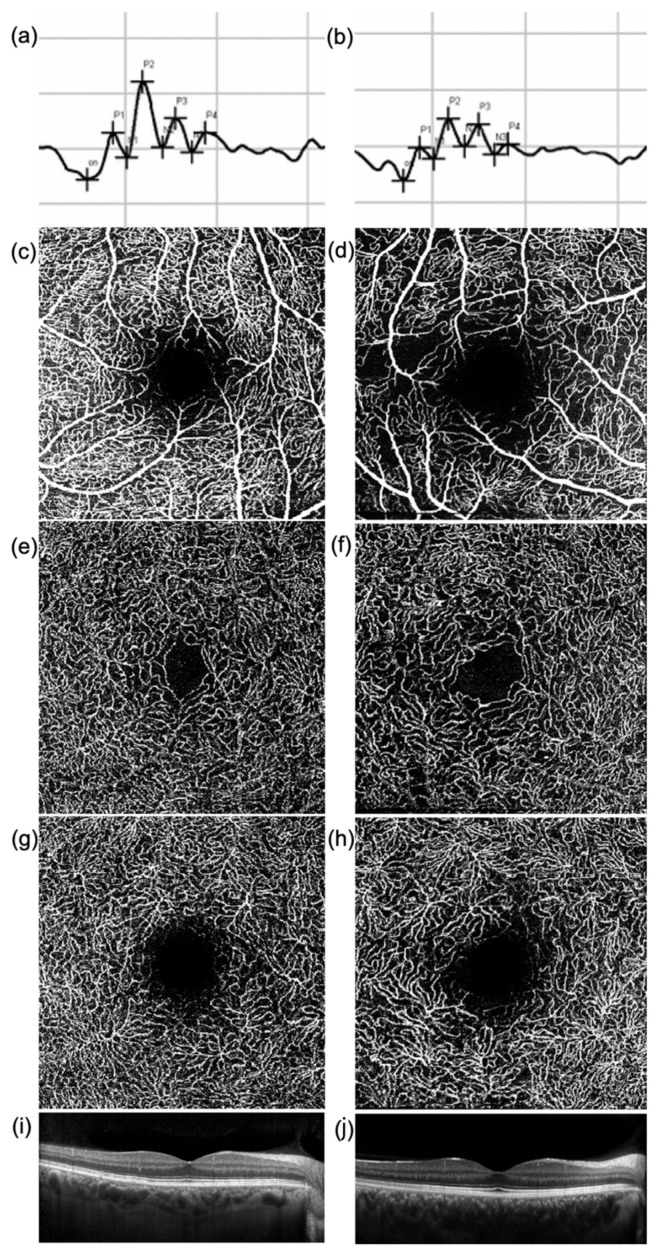
(**a**,**b**) Oscillatory Potentials (OP), (**c**–**h**) en-face optical coherence tomography (OCT) angiography images of (**c**,**d**) superficial vascular plexus (SVP), (**e**,**f**) intermediate (ICP), and (**g**,**h**) deep capillary plexus (DCP) and (**i**,**j**) OCT central B-scan of (**a**,**c**,**e**,**g**,**i**) a healthy subject and (**b**,**d**,**f**,**h**,**j**) a diabetic patient without diabetic retinopathy. The amplitude of each OP wave is reduced in the diabetic patient (**b**) compared to the healthy subject (**a**). The OCTA images show a rarefaction of the capillary networks in the diabetic patient (**d**,**f**,**h**) compared to the healthy subject (**c**,**e**,**g**), more evident in the SVP (**d**). No significant difference in the macular profile is evident at OCT (**i**,**j**).

As regards the OCTA results, no significant difference was found in the FAZ area of SVP, ICP, and DCP between the diabetic and the control group. OCTA vessels parameters of the SVP (VAD, VLF, and FD) were significantly reduced in the diabetic group versus the controls (*p*-value 0.003, 0.001, and 0.001, respectively). The VLF and FD of the ICP were both significantly reduced in the diabetic group versus the controls (*p*-value: 0.044 and 0.016, respectively). Similarly to the ICP, the VLF and FD of the DCP were both reduced in the diabetic group versus the controls (*p*-value: 0.058 and 0.024, respectively) (Figure 1).

### 3.2. Oscillatory Potentials

**Figure 2:**
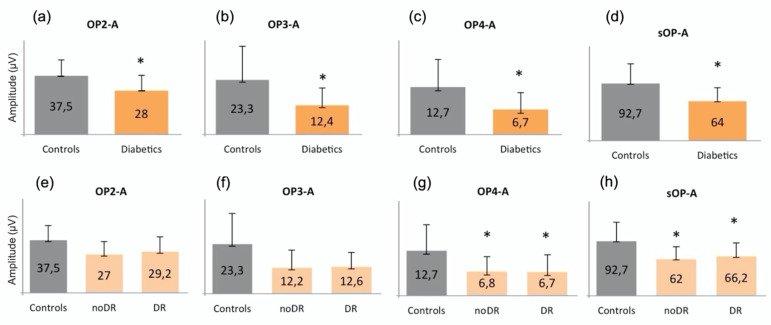
Graphical representations of oscillatory potentials in (**a**–**d**) controls compared to diabetic patients (Diabetics) and in (**e**–**h**) controls compared to subjects without (noDR) and with mild (DR) diabetic retinopathy. The asterisk (*) is used to identify statistically significant results versus the control group. All measurements are expressed in μV. The amplitude of (**a**) OP2, (**b**) OP3, (**c**) OP4, and (**d**) of the sum of all OPs is significantly reduced in the diabetic group compared to the controls. Moreover, the amplitude of (**g**) OP4 and (**h**) of the sum of all OPs is also reduced in noDR and in DR subjects compared to the controls. A: amplitude; OP: oscillatory potential; sOP: sum of oscillatory potentials; DR: diabetic retinopathy.

The amplitude of OP2, OP3, OP4, and sOP was significantly reduced in the diabetic group versus the controls (*p* = 0.376, *p* = 0.015, *p* = 0.011 and *p* = 0.013, respectively) (Figure 2a–d). In order to better understand the behavior of the different oscillatory potentials, we also analyzed patients without (noDR) and with mild DR (DR). We found that the A of OP4 and sOP was significantly reduced in both groups versus the controls (*p* = 0.041 and *p* = 0.049, respectively) (Figure 2g,h). No differences were found between noDR and DR subjects.

### 3.3. Correlations between Vascular Parameters and Oscillatory Potentials in Diabetics

For the SVP, a statistically significant direct correlation was found between the amplitude of OP1, OP2, OP3, and sOP and the VLF and FD. For the ICP, a statistically significant direct correlation was found between the implicit time of OP1 and OP2 and VDI, and a statistically significant inverse correlation was found between the amplitude of OP3 and VDI and between that of OP4 and VAD and VDI. For the DCP, a statistically significant inverse correlation was found between the implicit time of OP1 and VLF and FD, of OP2 and FD, and of OP4 and VAD, VLF, and FD, as well as between the amplitude of OP3, OP4, and sOP and VDI (Table 2).

## 4. Discussion

Retinal neurons and retinal vessels are both implicated in the pathophysiology of DR [15]. Using OCT, OCTA, and OP, the present study found that both retinal structural and vascular changes as well as functional ones are precociously present in diabetic eyes, compared to healthy controls, in accordance to previous reports, and that they are related to each other [14,19]. In particular, we found a reduction of VLF and FD of the intraretinal vascular networks at OCTA, representing the length and complexity of retinal microvessels, in diabetic patients with no or early signs of DR, as previously reported [14]. Moreover, we confirmed the presence of early OPs alterations in diabetic eyes compared to healthy controls. It is now known that the function of the neural components of the middle and inner retinal layers, detected by OP, is altered in patients with diabetes before the development of retinopathy. Thus, OP has been shown to be a sensitive measure of early neuronal abnormalities, long before DR can be clinically detected [15]. OPs are considered the most precociously altered functional parameter in diabetes, as confirmed in animal and human studies [11,20,21]. Different studies have analyzed the alterations of OPs in diabetic eyes with and without early DR. In particular, it has been frequently observed that the alterations primarily concern wave amplitudes, while the implicit times seem to be involved only later, with the progression of diabetic changes [13,16,21]. We found that the amplitude of OP2, OP3, OP4, and the sum of all OPs were significantly reduced in diabetic eyes, compared to the controls. Despite some controversies, OPs are thought to originate in the inner retinal layers, in particular, in the INL and IPL, where amacrine cells are specifically located, and are thought to represent a mainly negative modulatory feedback response of amacrine cells to ganglion and bipolar cells [11,22]. Moreover, in the present study, OP4 and the sum of all OP wavelets were reduced even in diabetic eyes without clinically detectable DR. Li et al. suggested the amplitude of OP4 as a good measure of early detection of retinal diabetic damage in diabetic patients without ophthalmoscopic signs of DR [21]. Among OP wavelets, OP4 physiologically shows the smallest amplitude, allowing an earlier detection of changes compared to the other wavelets. Our data confirm the early alterations of OPs secondary to diabetes, probably correlated with the precocious impairment of the complex neural crosstalk among retinal neurons, which are strictly interrelated, both morphologically and functionally, in the INL and IPL. One of the major regulators of this crosstalk is the amacrine cell [11]. Amacrine cells are characterized by a great variety of shapes, sizes, and stratification patterns, which are still under research [23,24]. Previous studies found that different cellular morphological subtypes present specific stimuli and responses even into the same microcircuit, with various synaptic and extrasynaptic receptors with differential sensitivity to neurotransmitters [25]. Recent studies in animal models on the amacrine cells AII and A17 demonstrated that diabetes causes different changes in the pharmacological properties and single-channel conductance of the synaptic receptors of these two cell populations, variably modifying their Ca2 permeability. This alteration of neuron and microcircuit dynamics occurred at a very early time point, earlier than several other functional changes and before microvascular manifestations [26,27]. Moreover, different murine diabetic models confirmed the precocious loss of amacrine cells compared to other neural cells in the retina [28,29,30]. One of the main and well-known characteristics of amacrine cells is their variable, but always significant, dendritic arborization, which mainly develops in the IPL and allows the complex function of integration and modulation of these interneurons that interact at the second synaptic level of vertically direct pathways [23]. Recently, it has been shown that, in ganglion cells, the first cellular structures to be damaged in diabetic patients seem to be cellular dendrites: this would suggest that dendrites may be the most precociously damaged elements in other neuronal cells, such as amacrine cells [24]. These data may suggest an early alteration of the mechanisms of connection and stimuli transmission among retinal neurons, explaining the precocious alteration of OPs [22].

OPs are thought not only to reflect the function of the inner and middle retina but also to be sensitive to changes in retinal circulation [11,15]. Recently, Ebihara et al. showed that OPs of the focal macular ERG are smaller with prolonged implicit times in association with a reduced macular vascular density in diabetic patients with no and mild/moderate DR [31]. However, as noted by the Authors, focal ERG analyzes only the macular cone-driven ERG component and does not record OP4. Luu et al. reported a significant negative correlation between OP4 amplitude, but no other OP components, and the caliber of retinal arterioles (using fundus photography) [15]. In our work, we studied patients with early diabetic retinal involvement (no moderate RD was included) without macular edema, using standard full-field ERG, not limited to the macula. We confirmed the correlation between OPs and OCTA parameters; moreover, we found that the first OP waves were related to a reduction of the length and complexity of intraretinal vessels (VLF and FD), mainly in the SVP, while OP3 and OP4 seemed to be related to changes in the caliber of retinal vessels (VDI), with an inverse correlation between the amplitude of both OP3 and OP4 (and the sum of all OPs) and the increase in vessel diameter in the deeper capillary plexuses (ICP and DCP), located at borders of the INL.

Our findings support the hypothesis that early retinal deep vascular dilatation and superficial vascular shortening may be correlated with a precocious neuronal dysfunction involving amacrine cells in diabetes [15,32]. It has also been suggested that changes in the retinal vascular caliber, now well detectable and quantifiable by OCTA, may reflect subclinical microvascular disease and provide prognostic information regarding the risk of retinopathy [15,32]. Our findings suggest that microvascular changes detectable by OCTA are also strictly related to early neurofunctional alterations, particularly involving the middle retina. OCTA is a recently developed imaging modality that allows the detection and quantification of intraretinal vascular parameters in a non-invasive way. It has been extensively applied in retinal diseases, particularly DR. However, the correlation between OCTA data and functional measurements is still matter of research, as well as the mechanisms of the complex neurofunctional intraretinal crosstalk in diabetes. Our study confirms the relevance of the interrelations between vascular and functional elements, namely OPs, in the very early changes of the diabetic retina, mainly involving the complex mechanism of neuronal feedback and regulations specifically modulated in the middle retina by amacrine cells. These cells interact with the retinal capillary plexuses maintaining their trophism and structure and have an important function in regulating the high metabolic and neuronal oxygen demands [5]. Some works have suggested that the alteration of OPs might be associated with a progression of the retinal disease, predicting worsening of DR [33,34]. Currently, we can also detect worsening of the vascular performance using OCTA to easily visualize the vascular plexuses.

The main limitations of this study are its cross-sectional design and the small number of enrolled patients, which may have limited the statistical significance of our analysis. However, we still found interesting correlations between morphological and functional parameters; future studies might further develop these data, particularly with longitudinal analyses, allowing the delineation of the time-related modifications of both functional and morphological vascular parameters. The understanding of retinal morpho-functional correlations in retinal diabetic disease is essential to identify new targets and the appropriate time of intervention.

In conclusion, this study detected a reduction of OPs, suggesting a precocious damage to amacrine cells, in diabetic eyes in the early phases of retinal involvement. Moreover, we found a correlation between OPs changes and OCTA parameters, confirming a relevant role of neurovascular crosstalk dysfunction in the development of DR and the ability of OCTA-specific parameters to detect early intraretinal vascular changes that represent early neuro-vascular diabetic dysfunction.

## Figures and Tables

**Table 1:** Characteristics of the study population.

	Control Group (9 Subjects)	Diabetic Group (22 Subjects)
Number of eyes	18	44
Mean age ± SD (years)	50.8 ± 12.9	50.5 ± 14.1
Sex (M:F)	4:5	12:10
Mean duration of DM ± SD (years)	-	15.2 ± 8.5
Type of DM (DM1:DM2)	-	7:15
Mean Intraocular tension ± SD (mmHg)	15.8 ± 1.3	16.8 ± 2
Mean refractive error ± SD (diopters)	−0.15 ± 1.31	−0.13 ± 1.02
Mean Visual acuity ± SD (ETDRS score)	85 ± 0	85 ± 0

M: male; F: female; SD: standard deviation; DM1, DM2: diabetes mellitus type 1, type 2; ETDRS: Early Treatment Diabetic Retinopathy Study.

**Table 2:** Correlations between OPs and OCTA vascular parameters.

	SVP	ICP	DCP
IT-OP1			
**VAD**	b = 0.004; *p* = 0.650	b = 0.004; *p* = 0.539	b = −0.016; *p* = 0.077
**VLF**	b = 0.001; *p* = 0.954	b = 0.001; *p* = 0.894	**b = −** **0.004; *p* = 0.042**
**VDI**	b = 0.039; *p* = 0.558	**b = 0.049; *p* = 0.043**	b = 0.005; *p* = 0.846
**FD**	b = −0.003; *p* = 0.632	b = 0.001; *p* = 0.967	**b = −0.0197; *p* = 0.030**
IT-OP2			
**VAD**	b = −0.003; *p* = 0.618	b = 0.001; *p* = 0.718	b = −0.008; *p* = 0.154
**VLF**	b = −0.001; *p* = 0.205	b = −0.001; *p* = 0.781	b = −0.002; *p* = 0.058
**VDI**	b = 0.040; *p* = 0.319	**b = 0.034; *p* = 0.019**	b = 0.016; *p* = 0.288
**FD**	b = −0.007; *p* = 0.080	b = −0.002; *p* = 0.659	**b = −0.011; *p* = 0.039**
IT-OP3			
**VAD**	b = −0.003; *p* = 0.448	b = 0.001; *p* = 0.891	b = −0.004; *p* = 0.361
**VLF**	b = −0.001; *p* = 0.211	b = −0.001; *p* = 0.737	b = −0.001; *p* = 0.204
**VDI**	b = 0.017; *p* = 0.593	b = 0.019; *p* = 0.105	b = 0.011; *p* = 0.355
**FD**	b = −0.005; *p* = 0.103	b = −0.002; *p* = 0.636	b = −0.006; *p* = 0.166
IT-OP4			
**VAD**	b = −0.001; *p* = 0.657	b = −0.002; *p* = 0.172	**b = −0.004; *p* = 0.023**
**VLF**	b = −0.001; *p* = 0.572	b = −0.001; *p* = 0.106	**b = −0.001; *p* = 0.009**
**VDI**	b = 0.001; *p* = 0.943	b = 0.001; *p* = 0.858	b = 0.001; *p* = 0.961
**FD**	b = −0.001; *p* = 0.411	b = −0.002; *p* = 0.092	**b = −0.005; *p* = 0.003**
A-OP1			
**VAD**	b = 0.002; *p* = 0.095	b = −0.001; *p* = 0.470	b = 0.001; *p* = 0.212
**VLF**	**b = 0.001; *p* = 0.002**	b = −0.001; *p* = 0.710	b = 0.001; *p* = 0.105
**VDI**	b = −0.011; *p* = 0.142	b = −0.004; *p* = 0.160	b = −0.002; *p* = 0.454
**FD**	**b = 0.002; *p* = 0.001**	b = −0.001; *p* = 0.771	b = 0.002; *p* = 0.082
A-OP2			
**VAD**	b = 0.001; *p* = 0.267	b = 0.001; *p* = 0.884	b = 0.001; *p* = 0.517
**VLF**	**b = 0.001; *p* = 0.033**	b = 0.001; *p* = 0.680	b = 0.001; *p* = 0.288
**VDI**	b = −0.006; *p* = 0.250	b = −0.002; *p* = 0.454	b = −0.003; *p* = 0.175
**FD**	**b = 0.001; *p* = 0.019**	b = 0.001; *p* = 0.567	b = 0.001; *p* = 0.162
A-OP3			
**VAD**	b = 0.002; *p* = 0.086	b = −0.001; *p* = 0.364	b = −0.001; *p* = 0.643
**VLF**	**b = 0.001; *p* = 0.002**	b = −0.001; *p* = 0.793	b = 0.001; *p* = 0.910
**VDI**	b = −0.011; *p* = 0.130	**b = −0.007; *p* = 0.013**	**b = −0.008; *p* = 0.005**
**FD**	**b = 0.002; *p* = 0.001**	b = −0.001; *p* = 0.923	b = 0.001; *p* = 0.837
A-OP4			
**VAD**	b = 0.001; *p* = 0.610	**b = −0.002; *p* = 0.050**	b = −0.001; *p* = 0.362
**VLF**	b = 0.001; *p* = 0.203	b = −0.001; *p* = 0.155	b = −0.001; *p* = 0.681
**VDI**	b = −0.011; *p* = 0.313	**b = −0.010; *p* = 0.009**	**b = −0.012; *p* = 0.008**
**FD**	b = 0.002; *p* = 0.185	b = −0.001; *p* = 0.190	b = −0.001; *p* = 0.775
A-OP			
**VAD**	b = 0.001; *p* = 0.125	b = −0.001; *p* = 0.435	b = 0.001; *p* = 0.756
**VLF**	**b = 0.001; *p* = 0.003**	b = −0.001; *p* = 0.775	b = 0.001; *p* = 0.397
**VDI**	b = −0.003; *p* = 0.123	b = −0.002; *p* = 0.055	**b = −0.002; *p* = 0.037**
**FD**	**b = 0.001; *p* = 0.002**	b = −0.001; *p* = 0.898	b = 0.001; *p* = 0.285

b: regression coefficient; P: *p*-value; IT: implicit time; A: amplitude; OP1-OP4: oscillatory potential waves; OP: sum of the PO waves; VAD: vessel area density; VLF: vessel length fraction; VDI: vessel diameter index; FD: fractal dimension. Statistically significant results in bold.

## Data Availability

The data presented in this study are available in the Article. Eventual additional data are available on request from the corresponding author.
